# Topology of evolving, mutagenized viral populations: quasispecies expansion, compression, and operation of negative selection

**DOI:** 10.1186/1471-2148-8-207

**Published:** 2008-07-17

**Authors:** Samuel Ojosnegros, Rubén Agudo, Macarena Sierra, Carlos Briones, Saleta Sierra, Claudia González- López, Esteban Domingo, Juan Cristina

**Affiliations:** 1 Centro de Biología Molecular "Severo Ochoa", UAM-CSIC. Campus de Cantoblanco, 28049, Madrid, Spain; 2 Laboratorio de Evolución Molecular, Centro de Astrobiología (CSIC/INTA), Instituto Nacional de Técnica Aeroespacial, Ctra de Torrejón a Ajalvir, km 4, 28850 Torrejón de Ardoz, Madrid, Spain; 3 Centro de Investigación Biomédica en Red de Enfermedades Hepáticas y Digestivas (CIBERehd), Spain; 4 Institute of Virology, University of Cologne, Fuerst-Pueckler Str. 56, D-50935 Cologne, Germany; 5 MRC Laboratory for Molecular Cell Biology & Cell Biology Unit, University College London, Gower Street, London, WC1E 6BT, UK; 6 Laboratorio de Virología Molecular, Centro de Investigaciones Nucleares, Facultad de Ciencias, Universidad de la República, Iguá 4225, 11400 Montevideo, Uruguay

## Abstract

**Background:**

The molecular events and evolutionary forces underlying lethal mutagenesis of virus (or virus extinction through an excess of mutations) are not well understood. Here we apply for the first time phylogenetic methods and Partition Analysis of Quasispecies (PAQ) to monitor genetic distances and intra-population structures of mutant spectra of foot-and-mouth disease virus (FMDV) quasispecies subjected to mutagenesis by base and nucleoside analogues.

**Results:**

Phylogenetic and PAQ analyses have revealed a highly dynamic variation of intrapopulation diversity of FMDV quasispecies. The population diversity first suffers striking expansions in the presence of mutagens and then compressions either when the presence of the mutagenic analogue was discontinued or when a mutation that decreased sensitivity to a mutagen was selected. The pattern of mutations found in the populations was in agreement with the behavior of the corresponding nucleotide analogues with FMDV *in vitro*. Mutations accumulated at preferred genomic sites, and dn/ds ratios indicate the operation of negative (or purifying) selection in populations subjected to mutagenesis. No evidence of unusually elevated genetic distances has been obtained for FMDV populations approaching extinction.

**Conclusion:**

Phylogenetic and PAQ analysis provide adequate procedures to describe the evolution of viral sequences subjected to lethal mutagenesis. These methods define the changes of intra-population structure more precisely than mutation frequencies and Shannon entropies. PAQ is very sensitive to variations of intrapopulation genetic distances. Strong negative (or purifying) selection operates in FMDV populations subjected to enhanced mutagenesis. The quantifications provide evidence that extinction does not imply unusual increases of intrapopulation complexity, in support of the lethal defection model of virus extinction.

## Background

RNA viruses replicate as mutant distributions termed viral quasispecies. This is a consequence of high mutation rates operating during RNA genome copying, due to the absence of proofreading-repair activities in the relevant RNA-dependent RNA polymerases and RNA-dependent DNA polymerases [1,2]. Most phylogenetic relationships among RNA viruses have been established using the consensus (or population) genomic sequences that represent a weighted average of multiple, closely related sequences present at each time point, in each virus sample obtained for analysis [3]. Phylogenetic relationships established with consensus viral sequences have been instrumental to classify viruses and to determine origin of emergent viruses and rates of virus evolution [1,4,5]. For many purposes it is important to analyze phylogenetically the relationship among different genomes from the same mutant spectrum of a viral quasispecies. This type of analysis may reveal the existence of genome subpopulations within mutant spectra that might encode different phenotypic traits. Also, it allows the calculation of average genetic distances among individual components of the mutant spectrum, a parameter that can be a predictor of biological behaviour [1]. As an example, a study with a poliovirus mutant which displays a -3-to 5-fold higher template-copying fidelity than the *wild type *documented that a narrow mutant spectrum impeded the virus to reach the brain of susceptible mice and produce neuropathology [6,7]. An early study documented that complexity of the coronavirus murine hepatitis virus quasispecies influenced the pathogenic potential of this virus for mice [8]. A broad hepatitis C (HCV) virus mutant spectrum was associated with poor response to treatment by ribavirin and interferon α [9], and rapid early evolution of the virus led to a chronic infection [10]. Some studies have found an association between a reduction of mutant spectrum complexity of HCV at early stages of treatment and viral clearance ([11]; reviewed in [12]). Recently, the role of the mutant spectrum in adaptation of West Nile virus has been documented [13,14]. Therefore, there is a need to develop methods to describe the relationship among components of mutant spectra in viral populations.

An estimate of the complexity and internal relationships among genomes within a mutant spectrum can be obtained through application of distance-based phylogenetic methods, such as neighbour-joining (NJ) [15] or maximum likelihood [16] procedures. However, the reliability of the derived genome clusters may be questionable when the number of mutations distinguishing different components of a mutant spectrum is small. Small genetic distances among genomes of a mutant spectrum are found when a viral clone (single genome) has undergone a limited number of passages in cell culture [17]. Despite this limitation, the general topology of a NJ tree may be robust enough to provide information about the evolutionary pattern undergone by the viral population.

An alternative method developed to group closely related sequences is Partition Analysis of Quasispecies (PAQ), which is considered a non-hierarchical clustering method [18]. PAQ groups together the viral sequences separated by the shortest genetic distances. For this purpose a centre genotype that nucleates a group of sequences within a circle (cluster) with a previously set radius is selected. High compactness of a group is defined by having a larger number of variants near the centre of the circle than at its boundary. In this fashion, multiple, overlapping or non-overlapping groups can be defined within mutant spectra of viral quasispecies. PAQ has been successfully used to analyze subpopulations of equine infectious anemia virus [19], Ty1-copia retrotransposon sequences [20], regulatory sequences of bovine viral diarrhoea virus [21], and sequential isolates of hepatitis A virus [22]. PAQ should find increasing application to describe viral quasispecies in view of its power to quantify relationships among related sequences, and of the critical role of mutant spectra in virus behaviour.

Our laboratory has studied quasispecies dynamics and evolution of foot-and-mouth disease virus (FMDV), a widespread picornavirus pathogen that causes the economically most important animal viral disease (reviews in [23,24]). The molecular epidemiology of FMDV has been analyzed in detail by establishing an extensive database of genomic nucleotide sequences, as well as by defining phylogenetic relationships among and within serotypes and subtypes of the virus [25-27]. FMDV has been used as a model system to investigate lethal mutagenesis, a term coined to describe virus extinction through an increase of viral mutation rate during replication ([28]; reviews in [29-31]). Studies on the interference that mutated FMDV and lymphocytic choriomeningitis virus (LCMV) populations exert on the infectivity of the corresponding standard genomes [32-35] led to the proposal of the lethal defection model of virus extinction [34]. According to this model, extinction can occur with a modest average number of mutations per genome that generates a class of genomes termed defectors, that can replicate but interfere with replication of the standard genomes [34,36]. Defector genomes differ from defective-interfering (DI) particles described for many viruses [37] in that DI particles are dependent on helper virus for replication while defectors are replication-competent [34,35].

In the course of the studies on lethal mutagenesis of FMDV, biological clones of the virus were subjected to serial cytolytic passages in cell culture in the absence or presence of the mutagenic bases or nucleoside analogues 5-fluorouracil (FU), ribavirin (1-β-D ribofuranosyl-1, 2, 3- triazole- 3 carboxamide) (R), or 5-azacytidine (AZC) [32,38-41]. These treatments have provided a number of viral populations differing in their mutant spectrum complexity, as measured by the average mutation frequency and Shannon entropy within mutant spectra [38-42]. However, possible alterations of phylogenetic relationships among components of the mutant spectra of mutagenized quasispecies have not been studied. To characterize possible changes among components of FMDV mutant spectra as a result of the mutagenic treatments, here we compare phylogenetic and PAQ analyses of several multiply-passaged FMDV populations, and their mutagenized counterparts. The results show that phylogenetic and clustering methods can provide a quantification of the mutagenic activity exerted on viral populations by reflecting changes in genetic distances among components of the mutant spectra of the viral quasispecies. PAQ analysis describes mutant distributions as spheres with size which is proportional to the genetic diversity, which varies as a result of enhanced mutagenesis. This type of representation has revealed that viral populations can respond rapidly to environmental changes, with striking switches between relaxation and compactness of the population diversity, that were not apparent from the comparison of mutation frequencies or Shannon entropies. Evidence is presented that identical or closely related consensus sequences may hide different subpopulations of genomes bearing distinct relationships among them.

## Methods

### Cells, viruses, and infections

The origin of BHK-21 cells, and procedures for cell growth, and infection of cell monolayers with foot-and-mouth disease virus (FMDV) have been previously described [39-41,43,44]. The following FMDV clonal populations have been used in the studies on lethal mutagenesis: i) FMDV C-S8c1, a plaque-purified derivative of the natural isolate C_1 _Santa-Pau Spain 70 [44], a representative of European serotype C FMDV [23], GenBank accession number NC_002554; ii) C-S8c1 multiply passaged at high multiplicity of infection (m.o.i.) in BHK-21 cells; the viral populations derived from each passage are indicated with a "p" before the passage number (i.e. C-S8p50 means C-S8c1 passaged 50 times in BHK-21 cells). iii) FMDV MARLS, a monoclonal antibody (mAb)-escape mutant, selected from population C-S8c1p213 [45], GeneBank accession number AF274010. iv) C-S8c1p260p3d, the viral population resulting from three diluted serial infections (mo.i. = 0.1) of C-S8c1p260 in BHK-21 cells [46]; gene bank accession number DQ409185; v) C^10^_280_, a clone derived from subjecting C-S8c1 to 280 plaque-to-plaque transfers in BHK-21 cells [47,48].

### Extraction of RNA, cDNA synthesis, PCR amplification and nucleotide sequencing

Procedures for extraction of RNA from the supernatants of infected cell cultures, reverse transcription (RT) of FMDV RNA and PCR amplification with high fidelity Pfu DNA polymerase have been previously described [32,39,40,48]. Molecular cloning in plasmids pET-28a3Dpol or pMT-28, as well as controls to ensure that molecular clones reflect accurately the composition of the quasispecies under analysis, were described in the primary publications that produced the genomic sequences analyzed here [46,49-52]. Nucleotide sequencing was performed using the Big Dye Terminator Cycle Sequencing Kit (Abi Prism; Applied Biosystems) and the automated sequencer ABI 373 and ABI 3730; all sequences were determined at least in duplicate, from independent sequencing reactions.

### Mutagenic agents and treatments

The procedures used for the mutagenic treatment have been described in detail in the primary references [30,33,38-40]. Here we provide a summary.

#### Treatment with ribavirin

**Figure 1:**
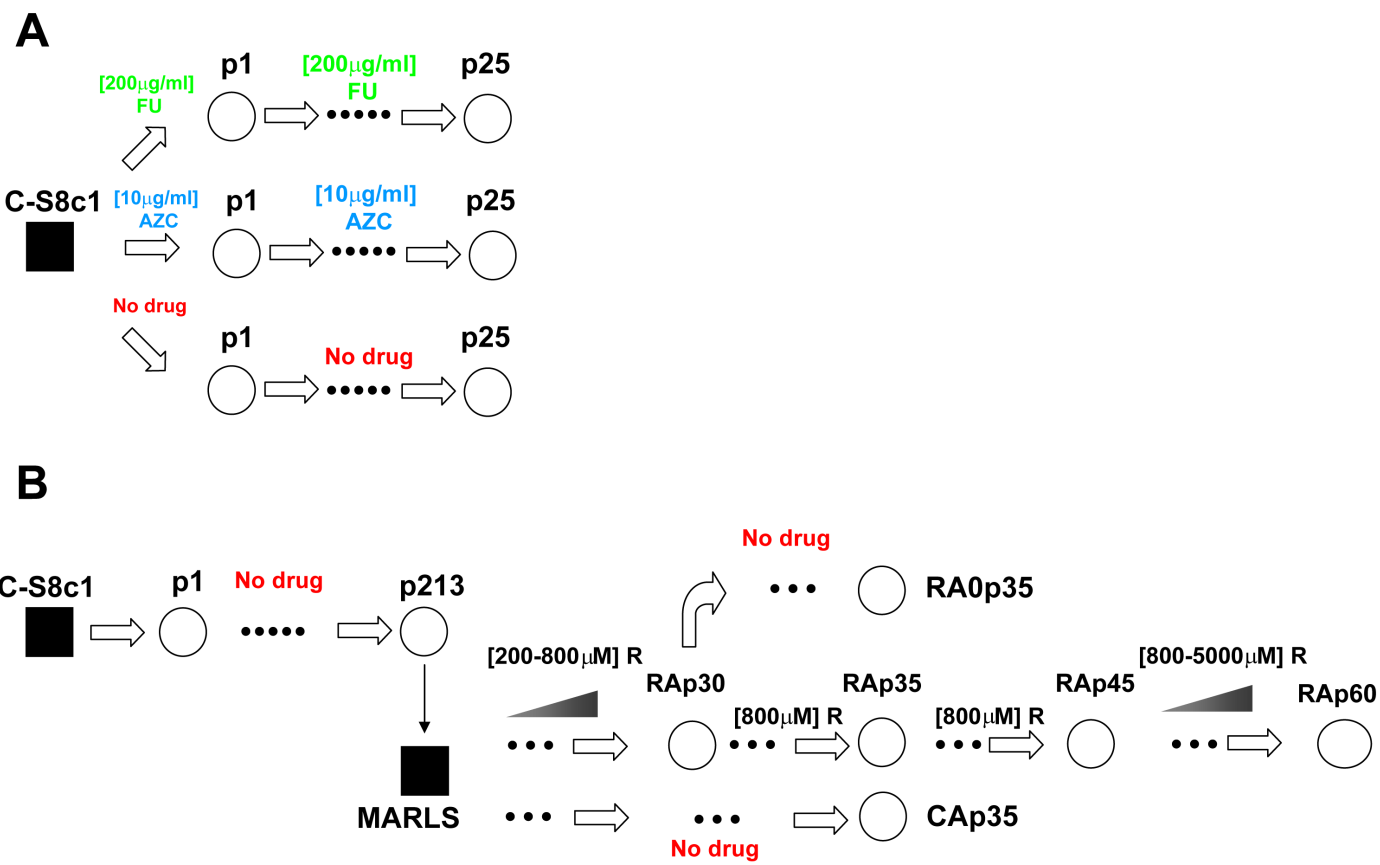
**scheme of passages of biological clone C-S8c1 in BHK-21 cells, in the presence or absence of mutagenic base and nucleotide analogues**. Biological clones are depicted as filled squares and uncloned populations as empty circles. Thick arrows indicate high m.o.i. passages (1 to 5 PFU/cell). Drug concentrations during the infections are indicated on the corresponding arrows. A) The initial biological clone C-S8c1 [44] was subjected to three parallel series of cytolytic infections, either in the presence of 5-fluorouracil (FU) or 5-azacytidine (AZC) or with no drug. B) C-S8c1 was subjected to 213 passages, and then MARLS clone was isolated as a mAb SD6 resistant mutant [45] (thin arrow). RA populations originated from serial cytolytic infections of MARLS in the presence of the indicated concentrations of ribavirin (R). The concentration of R was increased from 200 μM to 800 μM in the first 30 passages and from 800 μM to 5000 μM between passages 45 and 60. A bifurcation was established at passage 30 and the populations were subjected to 5 additional passages in the absence of R to yield the population RA0p35. CAp35 is the population obtained after 35 parallel passages of MARLS in the absence of drug.

A 100 mM solution of R (kindly provided by JC de la Torre) was prepared in PBS, sterilized by filtration, and stored at -20°C. Prior to use, the stock solution was diluted in DMEM (Dulbecco's modification of Eagle's medium) to reach the desired working concentrations (generally 200 μM to 5000 μM). Cell monolayers were preincubated with R for 7 h prior to infection. Infections in the absence of R, and mock-infected cells were maintained in parallel. FMDV MARLS was serially passaged in the presence and absence of increasing concentrations (200 μM to 800 μM) of R (Figure. 1). For each passage, 4 × 10^6 ^BHK-21 cells were infected with 1–4 × 10^6 ^PFU of virus from the previous passage until cytopathology was complete (about 30 h in the presence of R, and 16 h in the absence of R) [40,53].

#### Treatment with fluorouracil, azacytidine and guanidine hydrochloride

5-fluorouracil (FU) and azacytidine (AZC) were used as mutagenic base analogues (Figure 1), while guanidine hydrochloride (G) was used as inhibitor of FMDV replication, as previously described [38,39,41]. FU medium contained 200 μg/ml FU, and FUG medium contained 200 μg/ml FU and 4 mM G. Solutions were sterilized by filtration and stored at 4°C for a maximum of 15 days before use. Infections in the presence of FUG were performed as previously described [38,39,41].

The sources of all reagents for molecular studies are given in the corresponding references that describe the nucleotide sequences used in the present analyses [32,33,39,40].

#### Phylogenetic analysis

Multiple alignments of consensus sequences and molecular clones were carried out with the program CLUSTAL W [54], inserted into Bioedit package [55], using FMDV C-S8c1 and/or FMDV MARLS (GenBank accession numbers AJ133357 and AF274010, respectively) as the reference sequences. Pairwise distances matrix was generated by the program MEGA3.1 [56], using the Kimura-2 parameter model [57]. Tree topology was inferred by three phylogenetic methods: (i) Neighbor-Joining (NJ) [15] using also the MEGA3.1 package [56]; bootstrap re-sampling (1000 data sets) of the multiple alignments was used to test the statistical robustness of the trees obtained by NJ [58]. (ii) Maximum Parsimony (MP) (using the program DNAPARS from PHYLIP v3.5 package) [59]. (iii) Maximum Likelihood (ML) trees were generated by the program PUZZLE [16] using the Tamura-Nei substitution model [60], and the Gamma distributed rates with eight parameters (TN-8Γ) as heterogeneity model (see additional file 1). Additionally, for the additional file 2, a ML analysis of mutagenized populations of FMDV was performed by means of the program Modelgenerator [61], useful to identify the optimal evolutionary model (Akaike Information Criteria and Hierarchical Likelihood Ratio Test indicated that the GTR model best fit the sequence data). Using this model, ML trees were constructed using software from the PhyML program [62], available at [63]. As a measure of the robustness of each node, we used an approximate Likelihood Ratio Test (aLRT), which assesses that the branch being studied provides a significant likelihood gain, in comparison with the null hypothesis that involves collapsing the branch under study but leaving the rest of the tree topology unaffected [64].

#### Characterization of mutant spectra

The complexity of mutant spectra was quantified by minimum and maximum mutation frequencies. Minimum mutation frequency is the number of different mutations present in the molecular clones divided by the total number of nucleotides sequenced, and maximum mutation frequency is expressed as the total number of mutations present in the molecular clones divided by the total number of nucleotides sequenced. The normalized Shannon entropy (H), which is a measure of the proportion of identical sequences in a distribution [65], was also calculated. It is given by the formula:

(1) $$H=-[\Sigma_{i}^{N}(p_{i}\cdot \ln p_{i})]/\ln N$$

in which *p*_*i *_is the proportion of each sequence of the mutant spectrum, and N is the total number of sequences compared.

For the Partition Analysis of Quasispecies (PAQ) [18], the clones of each viral population were grouped together under the minimum possible radius, considering no partition. Within each cluster, the average distance (AD) value, with respect to the central sequence reported by PAQ, was calculated as a measure of the intrapopulation diversity, given by the formula:

(2) $$\text{AD}=\text{average distance}=(1/n)\Sigma_{i=1}^{n}(D_{ic})$$

in which *n *is the number of neighbors within the group with centre variant *c*, and *D*_*ic *_is the genetic distance (Hamming) between variants *i *and *c*. The populations are represented as spheres with the diameter proportional to the AD value. Standard errors have also been calculated (see additional file 3: Standard errors).

The nonsynonymous mutations corrected per nonsynonymous site (dn) and the synonymous mutations corrected per synonymous site (ds) were calculated using SNAP [66,67]. Kn and Ks are the ratio of nonsynonymous and synonymous mutations respectively, per nucleotide (without any correction). The sliding-window software K-Estimator [68] was used to infer the Ks and Kn in the FMDV genome using 200 nucleotide windows and a shift of 100 nucleotides per step, to cover all the polyprotein-coding region. The software calculates the confidence interval using Monte Carlo simulations.

Software for phylogenetic and sequence analyses were retrieved from the corresponding references listed in the different sections of Methods.

#### Statistical analysis

One Way ANOVA, Tukey post hoc tests for non equal n, and standard errors were calculated using Statistica 6.0 software package (StatSoft 2001).

## Results

### Phylogenetic evaluation of the mutagenesis-induced diversification of viral populations

The present study was aimed at analyzing retrospectively sets of consensus and clonal nucleotide sequences determined in our laboratory from FMDV populations subjected to serial cytolytic infections in BHK-21 cells, in the absence or presence of the mutagenic nucleotide analogues FU, R or AZC [32,33,39,40] – (Figure 1).

A representation of the quasispecies was obtained by determining the nucleotide sequences of 5 to 21 cDNA clones from each population, and scoring mutation types, mutation frequencies, and Shannon entropies. The FMDV genomic region analyzed was the 3D (polymerase)-coding region (Table 1). Phylogenetic trees were derived from the nucleotide clonal and consensus sequences from each population. Sequences from some reference viruses were also introduced as outgroups to establish minimal and previously known relationships, as well as to define a general structure of the tree. The distance-based NJ method [15], under Kimura two parameter model [57], was initially used for phylogenetic reconstructions, as described in Methods. In some cases the populations were analyzed using ML and MP algorithms [69] (see additional files 1 and 2). The general topology of these trees was consistent with that derived from the NJ analysis: the major clades and the relationships among clonal and consensus sequences were maintained.

**Table 1:** Number and type of mutations scored, and resulting mutation frequencies, calculated for the mutant spectra of the FMDV populations analyzed in the present study

Population^a^	Genomic residues^b^	Number of clones	Total nt. sequenced	Drug treatment^c^	Mutations^d^	Transitions^e^	Transversions^e^	Minimum mutation frequency (×10^-3^)^f^	Maximum mutation frequency (×10^-3^)^g^	Min/max ratio	H^h^	AD^i^
**No Drug p1**	6609–8013	5	7025	None	2/2	0.50	0.50	0.28	0.28	1	0.58	0.50
**AZCp1**	6609–8013	5	7025	[10 μg/ml] AZC	1/1	0	1	0.14	0.14	1	0.31	0.25
**FUp1**	6609–8013	5	7025	[200 μg/ml] FU	2/2	1	0	0.28	0.28	1	0.58	0.50
**No drug p25**	6609–8013	5	7025	None	1/3	1	0	0.14	0.42	0.33	0.61	0.75
**AZCp25**	6609–8013	18	25290	[10 μg/ml]AZC	13/19	0.21	0.79	0.51	0.75	0.68	0.73	2.31
**FUp25**	6609–8013	5	7025	[200 μg/ml] FU	14/19	1	0	1.99	2.7	0.74	1	6.75

**CAp35**	6667–7997	21	27951	None	17/17	0.88	0.12	0.61	0.61	1	0.69	0.85
**RA0p35**	7150–8020	21	18291	[800 μM] R, No drug	17/68	0.75	0.25	0.93	3.71	0.25	1	3.62
**RAp35**	6667–7997	14	18634	[500–800 μM] R	55/139	0.91	0.09	2.95	7.46	0.39	1	13.15 (7.85)
**RAp45**	6667–7997	12	15972	[800 μM] R	31/49	0.76	0.24	1.96	3.07	0.64	1	6.91
**RAp60**	6667–7997	14	18634	[800–5000 μM] R	55/80	0.97	0.02	2.95	4.29	0.69	1	7.62

^a^The origin of the FMDV populations is detailed in Methods and depicted schematically in Figure 1.

^b^The genomic residues analyzed correspond to the 3D-coding region of the FMDV genome; residue numbering is according to [47].

^c^Drug treatment (AZC, azacytidine; FU, 5-fluorouracil; R, ribavirin), and concentrations are as described in Figure 1.

^d^The first number indicates the total amount of different mutations found in all the clones analyzed; when repeated mutations were found the second number refers to the total number of mutations.

^e^Expressed per 1 in each population.

^f^Minimum mutation frequency is the number of different mutations divided by the total number of nucleotides sequenced.

^g^Maximum mutation frequency is the total number of mutations (including repeated mutations) divided by the total number of nucleotides sequenced.

^h^H is the normalized Shannon entropy [65].

^i^AD is the average genetic distance, calculated as detailed in Methods. The value in brackets was calculated with residues 7150–8020 (to compare with population RA0p35) of the FMDV genome.

**Figure 2:**
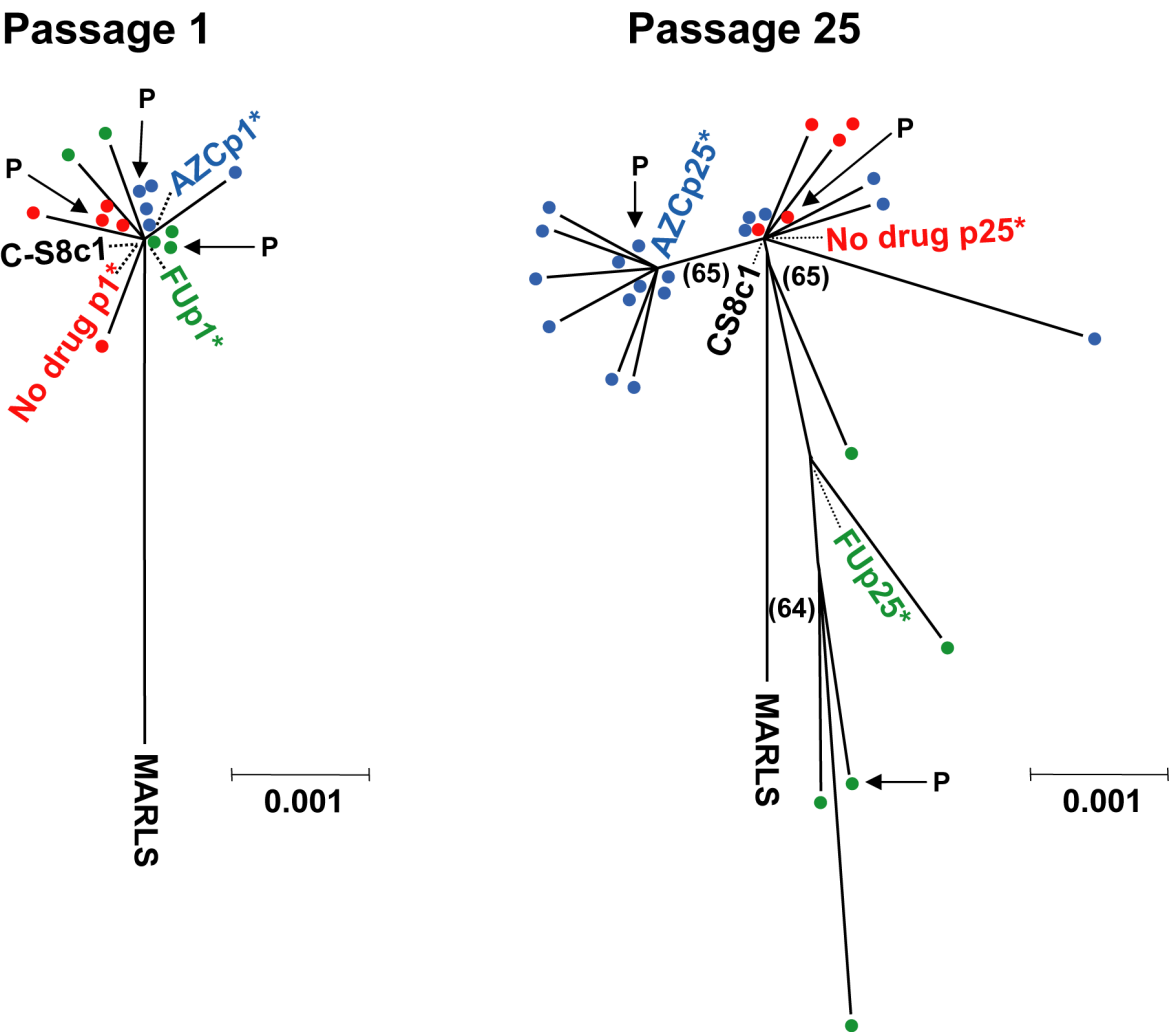
**phylogenetic analysis of individual molecular clones of FMDV populations passaged in the absence or presence of mutagens**. The trees were constructed with sequences from the 3D (polymerase)-coding region (nucleotide residues 6609 to 8013; numbering of residues as in [47]) of molecular clones obtained from populations at passage 1 (left) or passage 25 (right) of FMDV C-S8c1in BHK-21 cells. The populations analyzed correspond to those depicted in Figure 1A. Each dot represents the sequence of an individual clone as follows: red, clones from the populations passaged in the absence of drug (no drug); green, clones from the populations passaged in the presence of 5-fluorouracil (FU); blue, clones from the populations passaged in the presence of 5-azacytidine (AZC). The central Sequence according to PAQ is indicated by an arrow. The NJ method, with the Kimura 2 parameter algorithm, and 1000 bootstrap resamplings were used. Bootstrap values were below 75, and values higher than 60 are indicated in parenthesis. The position of the consensus sequence of the corresponding populations is indicated with an asterisk. MARLS was included as an outgroup of the tree. The origin of the viruses, and procedures for clonal analysis and nucleotide sequence determination are detailed in Methods.

Comparison of the phylogenetic trees derived from nucleotide sequences of the different FMDV clonal populations passaged 1 and 25 times in the absence or the presence of AZC or FU shows an expansion of genetic distances among components of the mutant spectrum of the mutated populations compared to the respective control populations passaged in the absence of drug. The presence of FU led to a higher expansion of the genetic distances than the presence of AZC (Figure 2). This expansion was not observed at passage 1 either with or without AZC or FU. Tree branches radiate from the corresponding consensus sequence with no discernible subclusters within each population.

**Figure 3:**
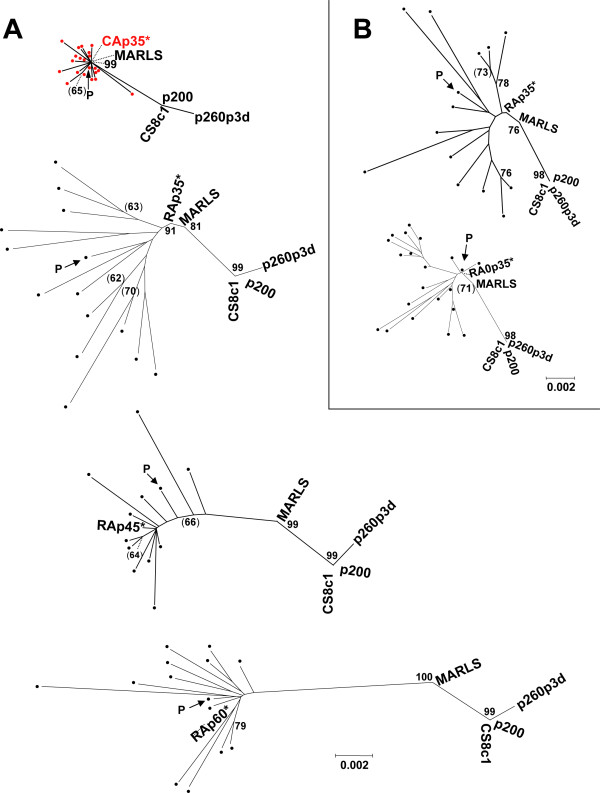
**phylogenetic analysis of individual molecular clones of MARLS populations passaged in the absence or presence of ribavirin**. Trees were constructed using the NJ method under Kimura 2-parameter model, with 1000 bootstrap resamplings. Bootstrap values higher than 75 are indicated with numbers; values between 60 and 74 are indicated in parenthesis. Each tree represents the analysis of one individual population from the MARLS lineage depicted in Figure 1B. Each population can be identified by the name of the corresponding consensus sequence indicated with an asterisk. Each dot represents the sequence of an individual molecular clone; red indicates absence of drug and black presence of ribavirin. The central Sequence according to PAQ is indicated by an arrow. A) Trees constructed with sequences from the 3D (polymerase)-coding region, residues 6667–7997, of FMDV. B) Comparison of the phylogenetic position of clones derived from populations RAp35 and RA0p35 (depicted in Figure 1); residues 7150–7997 from the 3D (polymerase)-coding were used. Procedures for clonal analysis and nucleotide sequencing are detailed in Methods.

A more complex pattern of intrapopulation genetic distances was observed with FMDV passaged in the presence of R (Figure 3). These populations originated from the high fitness FMDV clone MARLS [45]. The overall topology of the populations subjected to continuous mutagenic treatment (RAp35, RAp45 and RAp60; passage history depicted in Figure 1) shows the absence of significant subclusters. In turn, in all cases, a set of clones centrifugally diverge from the consensus sequence located at the basis of the group. As the number of passages in the presence of R increases, the genetic distance between the consensus sequence of each population and its parental MARLS clone increases substantially (about 11-fold from passage 35 to passage 60; Figure 3A).

Interestingly, the population that underwent 30 passages in the presence of R and then 5 passages in the absence of R, showed a more compact topology, and subclusters of clones were distinguished (RA0p35* in Figure 3B). It must be noted that, due to the close relatedness among the nucleotide sequences analyzed (see Background and Methods), the bootstrap values associated with the derived NJ trees suggest limited robustness of the derived clusterings. Nevertheless, the same general topological features are also observed when MP and ML were used (see additional file 1: neighbour-joining, maximum likelihood and maximum parsimony analysis of populations RAp35 and RA0p35) (see also statistical evaluation of PAQ, below).

The control population CAp35, obtained after 35 serial cytolytic infections of clone MARLS in cell culture in the absence of mutagen, shows a characteristic tree with evident lower diversity than the populations treated with mutagen (Figure 3), as documented by the very low mutation frequency value (12-fold smaller than population RAp35, see Table 1), and the average shortness of all the branches.

### Partition analysis of quasispecies (PAQ) applied to the clonal analysis of mutagenized FMDV populations

**Figure 4:**
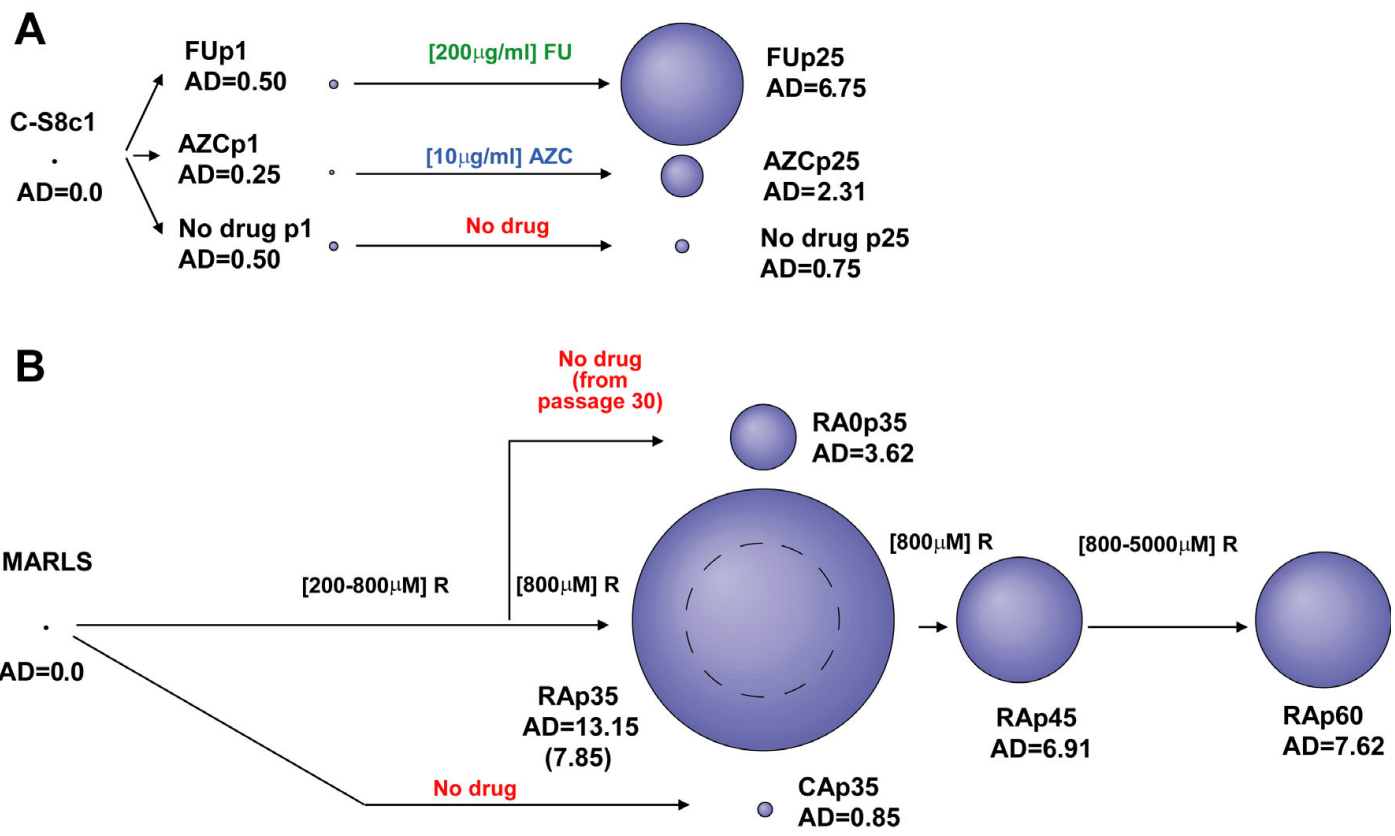
**partition analysis of quasispecies (PAQ) applied to sequences derived from mutagenized FMDV populations**. The initial biological clone of each passage series is depicted as a dot, with an initial intrapopulation average distance given arbitrarily the value AD = 0.0. The populations analyzed in A and B are those described in Figure 1A and Figure 1B, respectively. For simplicity, only the drug treatment (FU, 5-fluorouracil, green; AZC, azacytidine, blue; R, ribavirin, black; no drug, red), and the populations analyzed are depicted. The AD value was measured for each population considering no partition. Values are based on nucleotide sequences of the 3D(polymerase)-coding region, genomic residues 6609 to 8013 for A), and 6667 to 7997 for B), except for population RA0p35 for which residues 7150–7997 were used. For comparison, population RAp35 was also analyzed using residues 7150–7997; in that case the sphere is represented with a dotted line inside the large sphere. The diameter of each sphere is proportional to the AD value which is indicated next to each sphere. Procedures for nucleotide sequencing and PAQ analysis are described in Methods.

To further characterize the clonal structure of mutagenized FMDV populations, PAQ was applied to the same FMDV populations (Figure 1) studied by phylogeny. The sequences determined from the set of molecular clones derived from each viral population were treated as separate populations. A radius that grouped all sequences was chosen, and then the AD value was calculated (see Methods), as a measure of intrapopulation diversity (Figure 4). We considered the absence of intrapopulation partition as *a priori *realistic assumption because each viral population derives from a well defined ancestor and evolution took place under controlled conditions, supported also by phylogenetic data (Figure 3A).

PAQ analysis of the populations derived from clone C-S8c1 subjected to 25 passages either in the presence of FU or AZC or in the absence of mutagen, showed a 1.5-fold expansion of intrapopulation diversity as a result of virus propagation in the absence of mutagen, and 13.50-fold and 9.24-fold expansions as a result of passages in the presence of FU or AZC, respectively (Figure 4A). These values illustrate again the higher mutagenic potential of FU than AZC (Tukey post hoc test, p = 0.004) on FMDV replicating in BHK-21 cells [39].

A dramatic effect of R treatment was unveiled by PAQ through the comparison of MARLS populations passaged in the presence or absence of R (Figure 4B). Remarkably, multiple passages in the presence of up to 800 μM R, resulted in populations with significant differences in AD values by means of analysis of variance (One-way ANOVA: F = 85.5, df = 65, p < 0.001). RAp35 showed a 15.47-fold larger AD value (Tukey post hoc test, p < 0.001) than the population CAp35, the parallel control in the absence of the drug (Figure 4B).

The 3D of RAp35 and its derived populations harbours the substitution M296I which decreases its sensitivity to R [40]. Further passages in the presence of 800 μM R led to a reduction of diversity of population RAp45 (1.90-fold reduction in AD value, Tukey post hoc test, p < 0.001; Table 1). Once the diversity decreased in RAp45, it did not further increase significantly after 15 additional passages under higher (up to 5000 μM) concentrations of R (RAp60) (1.10 fold, Tukey post hoc test, p = 0.9).

Population RAp30 (depicted in Figure 1B) was subjected in parallel to 5 further passages either in the absence (RA0p35) or the presence (RAp35) of R. The intrapopulation diversity in RA0p35 was 2.15-fold smaller than RAp35 (Tukey post hoc test, p < 0.001) (see Discussion).

The modification of the relationship among components of the mutant spectrum is not reflected in Shannon entropy (which is saturated in the highest value, 1, for all the populations from the RA lineage tested) (see Table 1). Also maximum mutation frequency varies within a narrow range of 1.7-fold to 2.4-fold when the different populations are compared with RAp35 (table 1). There are no statistically significant differences in the minimum/maximum mutation frequency of population RAp35 and RA0p35 (one-way ANOVA: F = 2.57, df = 1.33, p = 0.118). This ratio is larger (higher diversity) in populations RAp45 and RAp60, than in population RAp35 (Tukey post-hoc test, p = 0.0073 and p = 0.013, respectively), despite having lower AD values and, therefore lower intrapopulation diversity. These comparisons support that the AD values derived from PAQ are a more sensitive and realistic descriptor of intrapopulation diversity than mutation frequencies.

### Mutational bias and rate of fixation of mutations

**Figure 5:**
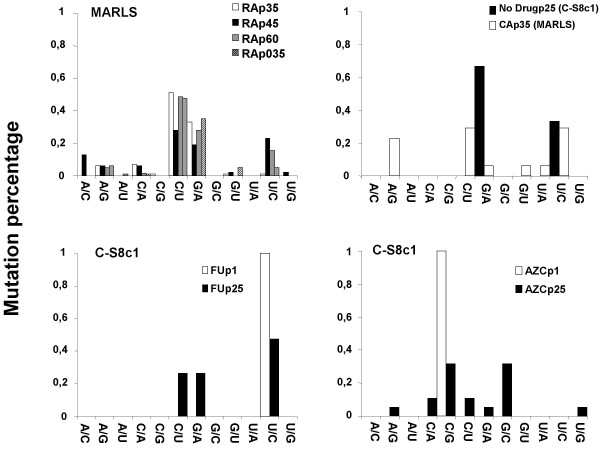
**proportion of the different mutation types scored among molecular clones of FMDV populations**. Each mutation type (abscissa) was counted for the indicated populations by comparing the nucleotide sequence from each individual molecular clone with the consensus sequence of the corresponding population. The populations analyzed are those derived from FMDV MARLS (top left panel), from populations passaged in the absence of drug and derived either from C-S8c1 or MARLS (top right panel), or from FMDV C-S8c1 (bottom panels). The populations correspond to those depicted in Figure 1, and can be identified according to the drug treatment (AZC, azacytidine; FU, 5-fluorouracil; R, ribavirin; no drug and C populations, control populations passaged in the absence of drug). Note the difference scale in ordinate of the top versus bottom panels. The genomic regions and number of nucleotides analyzed for each population are described in Table 1. Procedures for nucleotide sequence determination and bioinformatic analysis of sequences are detailed in Methods.

The mutation profile in the components of the mutant spectrum of each population analyzed indicated that a specific pattern of mutations was associated with each mutagen (Figure 5). FU-treated populations accumulated an excess of U → C transitions [39], while -RA populations preferentially fixed transitions C → U and G → A [42]. The mutational bias associated with AZC was mainly due to transversions G → C, C → G.

**Figure 6:**
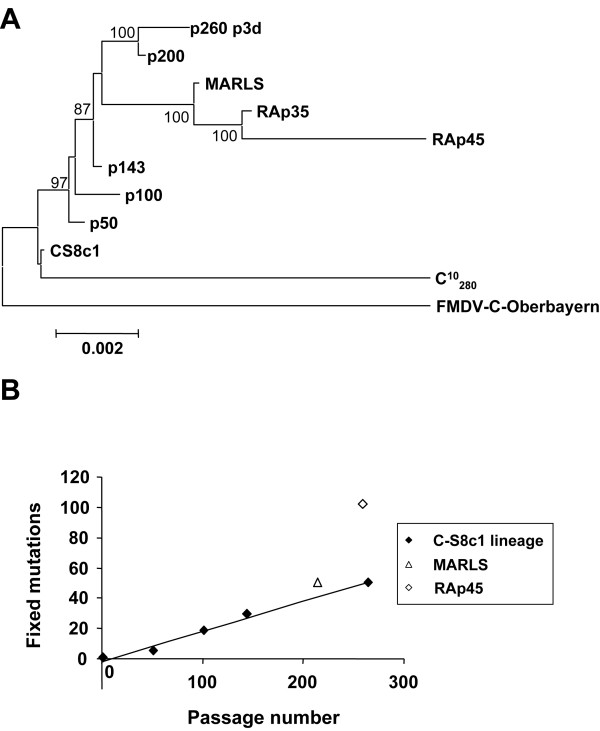
**quantification of the genotypic divergence of FMDV MARLS populations passaged in the presence of ribavirin**. The FMDV populations analyzed are those derived from C-S8c1 or MARLS, as displayed in Figure 1B. A) Phylogenetic tree based on consensus nucleotide sequences of populations derived from biological clone C-S8c1 (p50, p100, p143, p200) and from MARLS (RAp35, RAp45); p260p3d derives from C-S8c1 after 260 passages at high m.o.i. in BHK-21 cells, followed by 3 low m.o.i. infections, as described in [46]. C^10^_280_a is a clone of C-S8c1 subjected to 280 serial bottleneck (plaque-to-plaque) transfers in BHK-21 cells [47,48]. FMDV C-Oberbayern is a natural isolate whose sequence [26] has been used as outgroup. The tree is based on the nucleotide sequence of entire genomes, using the NJ method with 1000 bootstrap resamplings (nodes scoring values higher than 75 per 100 are indicated in the tree), following the procedures described in Methods. B) Absolute number of mutations including reversions, relative to the sequence of C-S8c1, as a function of passage number in the C-S8c1 lineage (depicted in Figure 1B). Values are based on the nucleotide sequence of entire genomes. A linear regression constructed with C-S8c1 and successive passages (C-S8c1 lineage) is shown (y = 0.1987x-1.6922; R^2 ^= 0.9882). The position of MARLS and RAp45 is indicated. Procedures are detailed in Methods.

The NJ tree derived from consensus nucleotide sequences of the entire FMDV genome of R treated populations disclosed an unusually rapid fixation of mutations in the consensus sequence of RAp45 (Figure 6A). In the course of serial cytolytic passages of C-S8c1, mutations accumulated at a rate of approximately 0.20 mutations per passage [48], with MARLS displaying a similar rate of 0.24 mutations per passage. In contrast, RA deviated to 1.13 mutations per passage, suggesting a very fast evolution associated with the presence of R (Figure 6B).

### Evidence of purifying selection during enhanced mutagenesis

**Figure 7:**
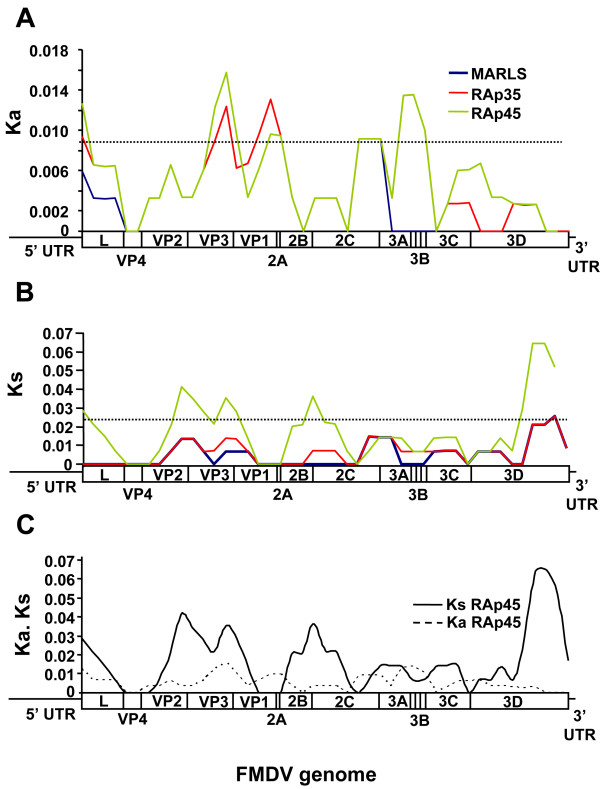
**distribution of Ka and Ks ratio (non-synonymous mutations per nucleotide, and synonymous mutations per nucleotide, respectively) along the consensus sequence of the protein-coding region of the genomes of FMDV MARLS, RAp35 and RAp45**. The origin of the populations analyzed is depicted in Figure 1B. The sequences have been compared with that of C-S8c1. Ka and Ks were calculated with the software K-estimator [68]. The genomes were analyzed in windows of 200 nt and a shift of the window of 100 nt in each step. The scaled protein-coding region of the FMDV genome is indicated in the abscissa [23,24]. The confidence intervals (dotted horizontal line) were calculated by k-estimator using Monte Carlo simulations [68]. A) Ka ratios. B) Ks ratios. C) Comparison of Ka and Ks ratios for RAp45.

We have examined whether the highly mutated RA genomes resulted from random fixation of mutations along the genome, or from their preferential accumulation at specific genomic sites. Using the sliding-window-based software K-estimator [68] (described in Methods) we measured the distribution of mutations (Ka, Ks, nonsynonymous and synonymous mutations per nucleotide, respectively) along the open-reading frame (polyprotein-coding region) of MARLS, RAp35 and RAp45 populations. At certain genome regions, Ka and Ks for RAp35 and RAp45 displayed values that were statistically significantly higher than the average (Figure 7). The asymmetric distribution of mutations suggests that despite the high mutational pressure imposed by R treatment, some kind of selection is still acting during the -passaging of the virus with error-prone replication.

To distinguish whether the asymmetric distribution of mutations along the genome was mainly due to positive or negative (or purifying) selection, the dn/ds ratios were calculated (see Methods and Table 2). -RA populations included 6- to 14- fold excess of synonymous mutations, both in the analysis of the mutant spectrum and the consensus sequence. In contrast, the dn/ds ratio of the consensus sequence of C-S8c1 at passage 263 (C-S8p260p3d) was 0.62, that is, 6-to 8-fold higher than the value for -RA populations. Also, a set of MARLS-derived clones scored a dn/ds ratio of 1.21, 7- to 17-fold higher value than obtained in the clonal analysis of -RA populations (Table 2). These data, together with the non-random accumulation of mutations along the viral genome (Figure 7), strongly suggest that purifying selection operates on FMDV in the course of replication under enhanced mutagenesis.

**Table 2:** Corrected ratio of nonsynonymous to synonymous substitution in several FMDV populations

Viral population^a^	dn/ds (Molecular clones)^b^	dn/ds (Consensus sequences)^c^
RAp35	0.16	N.D
RAp45	0.08	0.09 (MALRS)
RAp60	0.07	0.08 (MARLS)
RA0p35	0.12	N.D.
C-S8p260p3d	N.D.	0.63 (C-S8c1)
MARLS	1.21^d^	0.65 (C-S8c1)

N.D. not determined.

^a^The viral populations analyzed are those described in Figure 1 and Methods.

^b^dn/ds is the ratio of nonsynonymous mutations (corrected per nonsynonymous site) to synonymous mutation (corrected per synonymous site), calculated as described in Methods [85,86]. Values correspond to calculations using nucleotide sequences from molecular clones (as described in Table 1).

^c^dn/ds calculated as in b, applied to consensus nucleotide sequences, compared with the corresponding parental clone (given in parenthesis).

^d^This value was calculated with biological clones derived from MARLS, using the L (leader) protease-and capsid-coding regions.

### Virus extinction without a large excess of mutations

**Figure 8:**
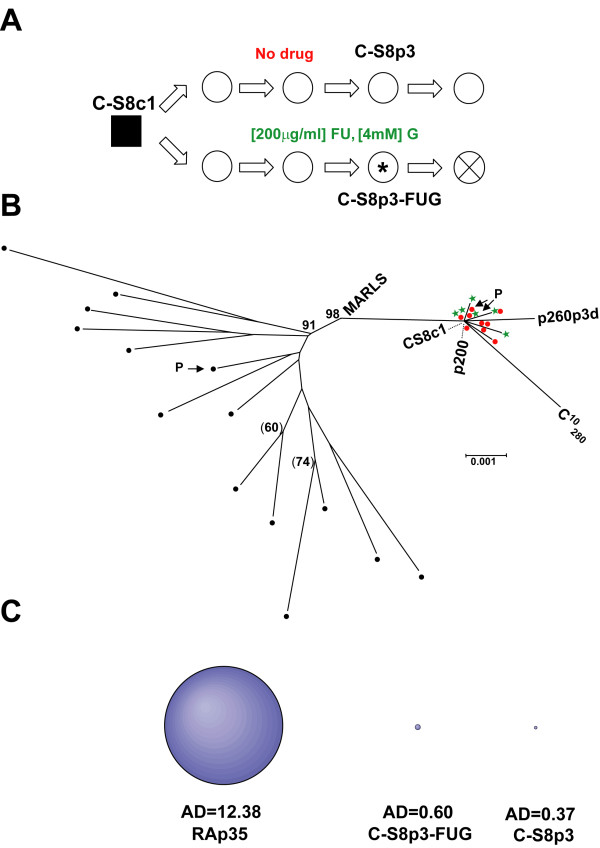
**preextinction FMDV population, and phylogenetic and PAQ analysis of mutagenized populations**. A) Biological clones are depicted as filled squares and uncloned populations as empty circles. Thick arrows indicate high m.o.i. passages (1 to 5 PFU/cell). Drug concentrations during the infections are indicated on the corresponding arrows). The circle with a cross represents an extinct population. * This is the last infectious population recovered. B) Phylogenetic analysis of individual molecular clones from a MARLS population passaged in the presence of R, and clones from the C-S8c1 lineage passaged in the presence or absence of FU and G. Black dots in the tree represent sequences of individual molecular clones from population RAp35. The tree includes also clones derived from the population obtained after 3 passages of C-S8c1 in the presence of FU and G (green asterisks), or in the absence of drugs (red dots). The central Sequence according to PAQ is indicated by an arrow. Populations p200, p260p3d and clone C^10^_280_, are derived from C-S8c1, as described in Figure 6. The tree is based on residues 6670 to 7871 of the 3D (polymerase)-coding region, using the neighbor-joining method (1000 bootstrap resamplings; values higher than 75 are indicated with numbers; values between 60 and 74 are indicated in parenthesis), as detailed in Figure 2 and Methods. C) AD values and spheres corresponding to the PAQ analysis of populations RAp35, C-S8p3-FUG and C-S8p3. Procedures are detailed in Methods.

To investigate whether FMDV extinction by enhanced mutagenesis was associated with unusual intrapopulation divergence [29,30,70,71], a PAQ analysis was performed on populations FMDV C-S8p3, C-S8p3-FUG and RAp35 [72] (Figure 8A). Preextinction population C-S8p3-FUG manifested a very modest increase in mutant spectrum complexity relative to the control population C-S8p3 (Figure 8C). Furthermore, the complexity of C-S8p3-FUG was much lower than the complexity of RAp35 (AD value comparison: T student, t = 12.19, p < 0.001), included in the phylogenetic and PAQ analyses (Figure 8B and 8C). Therefore, viral extinction can be achieved without any salient increase of the average genetic distances among components of the quasispecies (see Discussion).

## Discussion

Several remarkable modifications in the mutant spectrum occur when viral populations replicate in the presence of mutagenic agents such as base or nucleoside analogues. The initial experiments by J.J. Holland and colleagues documented very modest (1.1- to 2.8-fold) increases of mutation frequency at single base sites of poliovirus and vesicular stomatitis virus, associated with severe decrease of infectivity, as a consequence of enhanced mutagenesis by a variety of mutagenic agents [73]. Subsequent work showed that replication of different viruses in the presence of mutagenic base or nucleoside analogues could lead to virus extinction accompanied of very modest (from barely measurable up to 6.4-fold) increases of mutation frequency and mutant spectrum complexity ([28,39,74]; reviews in [29,30,71]). In our previous studies with FMDV and lymphocytic choriomeningitis virus we have observed that in the course of the transition of the viruses towards extinction: i) there is a 10^2^- to 10^3^-fold decrease in specific infectivity (number of infectious units divided by the total amount of viral RNA); ii) low viral loads and low replication capacity (fitness) favour extinction; and iii) internal, interfering interactions among mutagenized components of the mutant spectra play an important role in the extinction [32-36,38,39,75].

The critical participation of the mutant spectrum in viral extinction led to the proposal of "lethal defection" as a model of virus extinction by lethal mutagenesis [34,35], and motivated the phylogenetic and partition analyses of mutant spectra reported in the present study. The results support our previous conclusions on the general increase of mutant spectrum complexity -here documented by average genetic distances in phylogenetic trees and quantified by the AD parameter in PAQ- as a result of the various mutagenic treatments. Moreover, new insights into the events associated with enhanced mutagenesis have been unveiled by the comparison of phylogenetic and PAQ analyses. The NJ tree topology of a population evolved under enhanced mutagenesis conditions consists of a basal consensus sequence, with the individual components of the population radiating from it (the more mutated the population the bigger the radiation of the branches) and with no apparent population structure in the form of subclusters. Trees constructed with FMDV populations whose 3D (polymerase) included a substitution that decreased the sensitivity to R (RAp45 and RAp60) [40] displayed a slight reduction of branch length, but maintained the same general topology. This is in sharp contrast with population RA0p35, which, after only 5 passages in the absence of R, presents an internal structure completely rearranged, as shown by the NJ tree, and confirmed with ML and MP methods (Figure 3B, and additional file 1: neighbour-joining, maximum likelihood and maximum parsimony analysis of populations RAp35 and RA0p35).

The average distance parameter AD of PAQ reflects the fine detail of the intra population structure of diversity better than the other estimators traditionally used, such as mutation frequencies or Shannon entropy. Mutation frequency does not capture the distribution of mutations among the individual components of a mutant spectrum, and the Shannon entropy reaches the maximum possible value of 1 when all the sequences under study are different, independently of the mutational load. This lack of resolution is circumvented by PAQ due to the pairwise comparison of all the clones with respect to the central sequence. In this sense, any subtle variation in the spatial distribution of mutation frequency may be amplified yielding a more sensitive, accurate and non-saturating description of the internal structure of the population. The present analysis has documented striking expansions of the average intrapopulation genetic distance associated with mutagenic treatments (Figure 4). Interestingly the analysis of the RA lineage revealed that, after an initial expansion of intrapopulation diversity, a remarkably fast contraction of the average distance occurred in two situations. First, a 2.15-fold reduction in AD value was observed when the drug treatment was discontinued for only 5 passages. Second, populations with FMDV including in its 3D (polymerase) replacement M296I -that decreases the sensitivity to R [40] – displayed a 1.90-fold reduction in AD value (RAp45 and RAp60 populations, Figure 4). The ratio of minimum over maximum mutation frequency yielded higher values (indicative of higher diversity), in populations RAp45 and RAp60 than in population RAp35 which is the most diverse. Therefore mutation frequencies (both maximum, minimum or their ratio) and Shannon entropy, are less definitory than PAQ (when analyzing AD) to characterize the internal genetic diversity of mutagenized viral populations.

A possible interpretation of intrapopulation contractions is that in the course of the mutagenic treatment subsets of genomes with better than average replication efficiency are produced. However, their potential replicative advantage can not be expressed due to the continuous mutational input due to the presence of R. When R pressure is removed, specific subsets of genomes showing higher than average replication capacity, replenish the population. A similar, albeit less pronounced, contraction would occur as a consequence of the dominance of viral mutants with decreased sensitivity to R.

The MARLS-derived populations that replicated in the presence of R (the RA lineage, Figure 1B), showed unexpected high resistance to extinction at passage 35 and subsequent passages, associated with a mutation that decreased the sensitivity to R [40]. Despite such mutation, the number of total mutations fixed in the consensus sequence of population RAp35 was very high (1.13 mutations/passage, Figure 6) when compared with its ancestor (0.2 mutation/passage). Also the molecular clones derived from RAp35 presented a 12-fold increase in mutation frequency with respect to the control population, CAp35 (Table 1). The specific pattern of mutations of FU-treated, AZC-treated and R-treated populations (Figure 5), and the increase in mutant frequency with respect control populations (Table 1) suggest that the increase in mutation frequencies was effectively produced by the action of mutagens, in agreement with the mutational bias induced in the course of polymerization assays in the presence of R-triphosphate or FU-triphosphate using purified FMDV 3D (polymerase) ([40], Agudo et al submitted for publication).

Despite the high error frequencies induced by mutagen, mutations did not accumulate at random along the viral genome, but rather they accumulated at preferred genomic regions (Figure 7). The non-random accumulation of mutations and the excess of corrected synonymous mutations (Table 1) are consistent with purifying selection operating in the evolution of mutagenized populations. Viruses harbouring less deleterious mutations might have a selective advantage in a landscape of highly damaged viruses. In this view, the quasispecies would display a mutagenesis buffering activity, accepting those mutations that affect the more permissive regions of the viral genome. This dynamics is strikingly parallel to that observed in the course of bottleneck transfers carried out with the same FMDV clones C-S8c1 ([47,48,76-78] reviewed in [79]). In bottleneck transfers individual clones are selected for replication. In this situation Müller ratchet effect operates on the clones [80]. In clones that resisted extinction, mutations accumulated preferentially at some genomic regions and, again, an excess of synonymous mutations was found [48,77].

The present study supports the "lethal defection" model of viral extinction in that a limited number of mutations per genome can be sufficient to drive a virus to extinction [28,34,74]. Again, the comparison with FMDV clones subjected up to 409 bottleneck transfers is highly significant. In such clones extinction was not achieved despite the genomes reaching average mutation frequencies which are 5- to 37-fold higher than those associated with viral extinction by lethal mutagenesis [32,39,41,48]. It has been proposed that in successive bottleneck transfers, the kinetics of mutation accumulation allows the fixation of compensatory beneficial mutations that counteract the Müller Ratchet effect [76-79].

Several theoretical models of lethal mutagenesis of viruses have been proposed, either as a direct consequence of quasispecies dynamics and its corollary concept of error catastrophe, or independently of error catastrophe [70,81-84]. All models converge in that lethal mutagenesis is a feasible strategy to achieve virus extinction by mutagenic nucleotide analogues. What the models did not take into consideration is the key influence on extinction of internal interactions exerted among components of the mutant spectra. Lethal and interfering mutations impede a substantial "evaporation" (or "diffusion") of genomic sequences in sequence space [84], as it could not be otherwise, considering that we are dealing with loss of multiple biological functions compactly integrated in a viral genome [70]. The phylogenetic and PAQ approaches used here should be extremely helpful to monitor in a quantitative fashion the evolution of mutagenized viral populations both in cell culture and in vivo, as they increase their mutational load and either succumb or escape extinction.

## Conclusion

Phylogenetic and PAQ analyses have unveiled changes in the internal population structure of FMDV viral quasispecies subjected to mutagenesis by base and nucleoside analogues. Expansions and compressions of mutant spectra have been quantitated by comparing average genetic distances among components of mutant spectra. Comparisons of the types and distribution of mutations along the viral genomes have shown that negative (or purifying selection) acts in the course of enhanced mutagenesis. Virus extinction can be achieved with modest increases of population complexity. The average distance parameter (AD) reflects the intrapopulation structure better than the other estimators traditionally used, such as mutation frequencies or Shannon entropy.

## Authors' contributions

RA, MS, SS and CG-L determined nucleotide sequences. SO, CB and JC applied phylogenetic and PAQ methods. SO made the calculations. ED conceived the study, and SO and ED wrote the manuscript. All authors reviewed and approved the final manuscript.

## Supplementary Material

Additional file 1 **Neighbour-joining, maximum likelihood and maximum parsimony analysis of populations RAp35 and RA0p35**. Strains in the tree are shown by name. Consensus sequence of each population is indicated with an asterisk. Bar at the bottom of the trees denote distance A) and D) Neighbour joining trees with Kimura 2-parameter. Bootstrap resampling values (1000 replicas) higher than 50 are shown in red in the tree. B) and E) Maximum likelihood trees constructed with the Tamura-Nei substitution model and the Gamma distributed rates with eight parameters (TN-8Γ) as heterogeneity model. C) and F) Maximum parsimony trees, confidence values higher than 50 are shown in red in the tree. Click here for file

Additional file 2 **Maximum likelihood phylogenetic analysis of mutagenized populations of FMDV**. Strains in the tree are shown by name. Bar at the bottom of the trees denote distance. Consensus sequence indicated by *. The model used was GTR [61]. As a measure of the robustness of each node an approximate Likelihood Ratio Test was used. Click here for file

Additional file 3 **Standard errors**. Average Hamming distance of the components of each population (with respect to the central sequence selected by PAQ). Standard errors are plotted with boxes around the mean, as depicted in the legend. Click here for file
