# Supplementary figures and images for: Topology of evolving, mutagenized viral populations: quasispecies expansion, compression, and operation of negative selection
Source: BMC Evol Biol. 2008 Jul 17;8:207. doi: 10.1186/1471-2148-8-207 (PMC2515104; doi:10.1186/1471-2148-8-207)

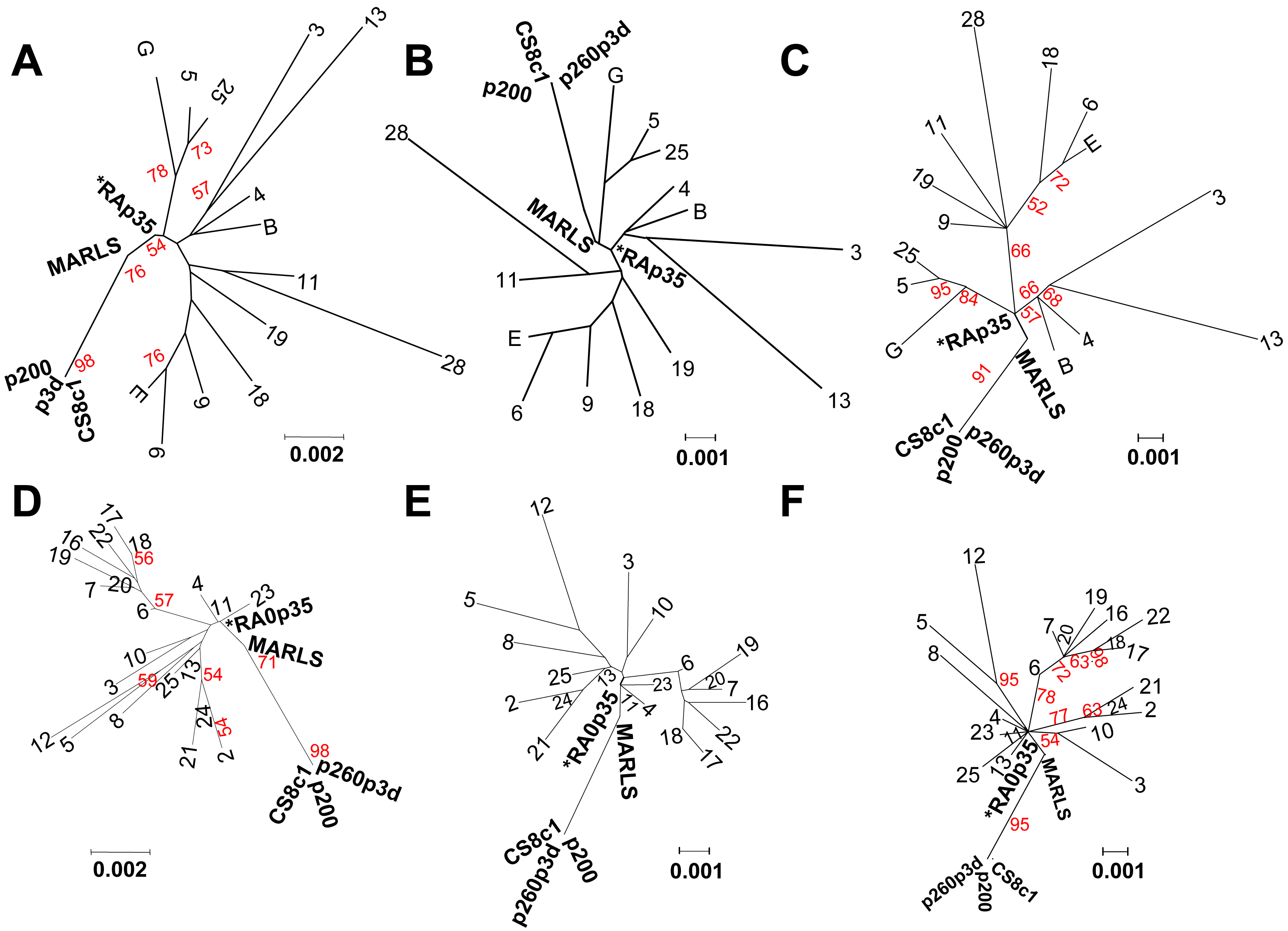

Supplement: Additional file 1 — Neighbour-joining, maximum likelihood and maximum parsimony analysis of populations RAp35 and RA0p35. Strains in the tree are shown by name. Consensus sequence of each population is indicated with an asterisk. Bar at the bottom of the trees denote distance A) and D) Neighbour joining trees with Kimura 2-parameter. Bootstrap resampling values (1000 replicas) higher than 50 are shown in red in the tree. B) and E) Maximum likelihood trees constructed with the Tamura-Nei substitution model and the Gamma distributed rates with eight parameters (TN-8Γ) as heterogeneity model. C) and F) Maximum parsimony trees, confidence values higher than 50 are shown in red in the tree. [file 1471-2148-8-207-S1.tiff]

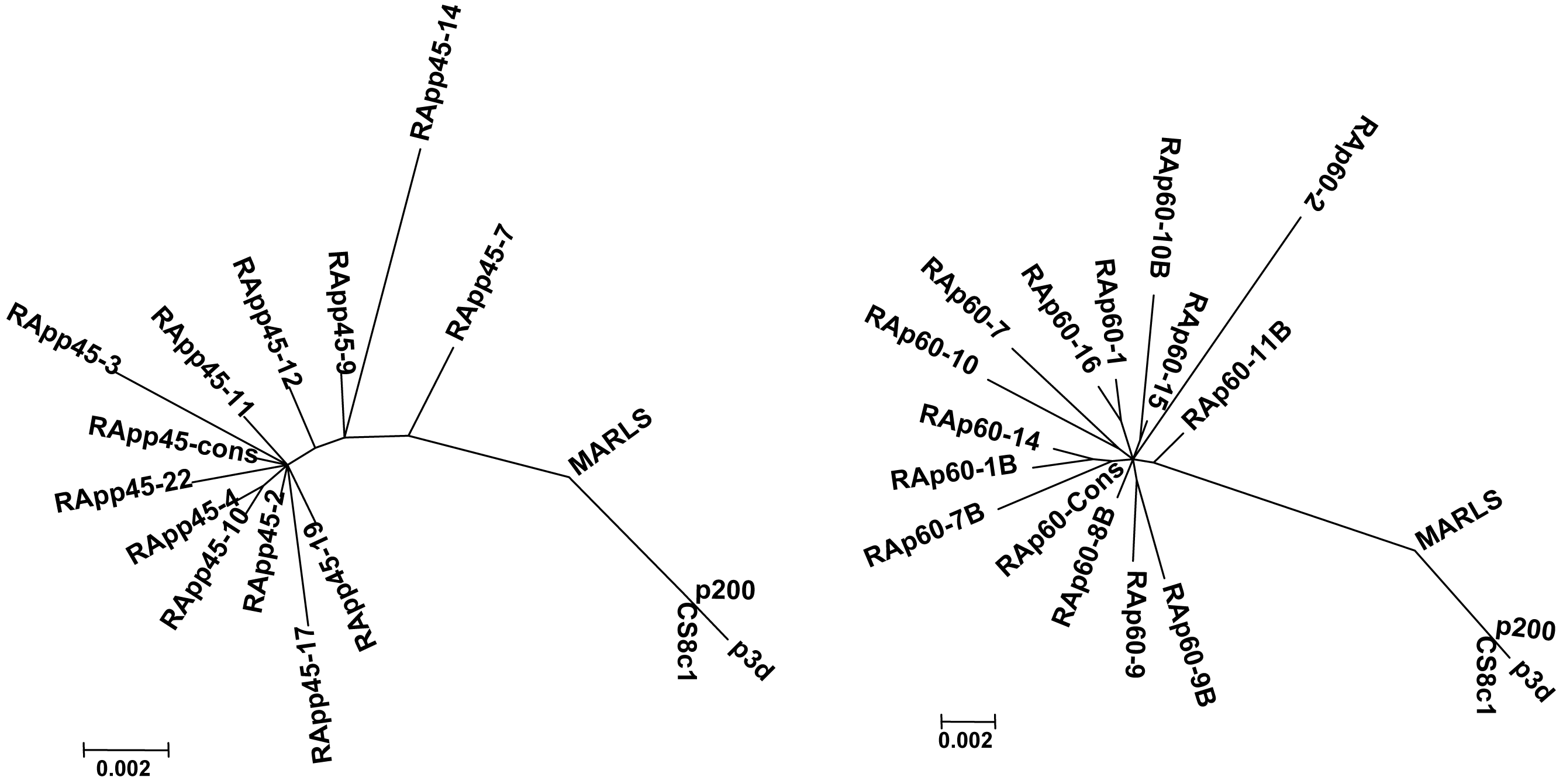

Supplement: Additional file 2 — Maximum likelihood phylogenetic analysis of mutagenized populations of FMDV. Strains in the tree are shown by name. Bar at the bottom of the trees denote distance. Consensus sequence indicated by *. The model used was GTR [61]. As a measure of the robustness of each node an approximate Likelihood Ratio Test was used. [file 1471-2148-8-207-S2.tiff]

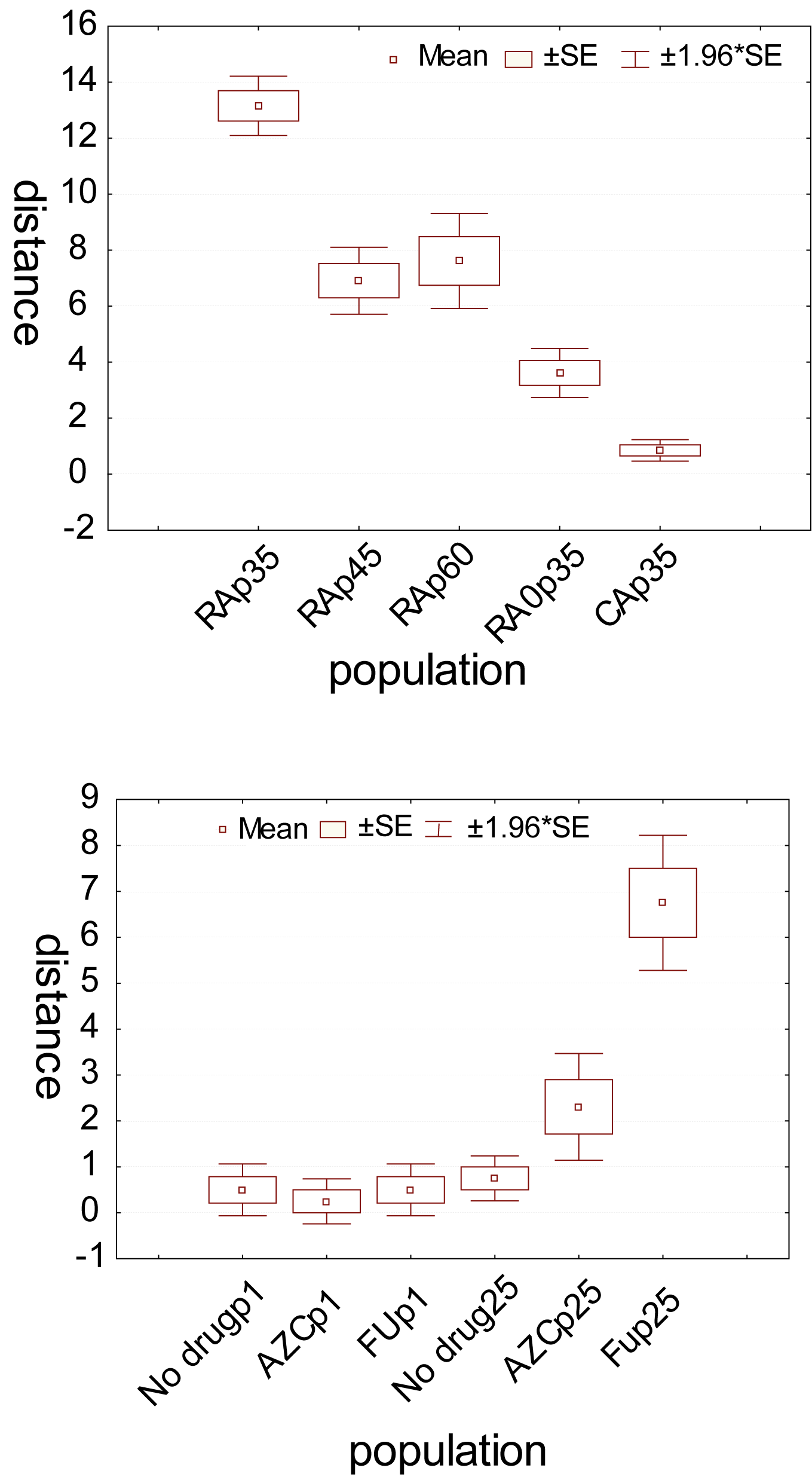

Supplement: Additional file 3 — Standard errors. Average Hamming distance of the components of each population (with respect to the central sequence selected by PAQ). Standard errors are plotted with boxes around the mean, as depicted in the legend. [file 1471-2148-8-207-S3.tiff]
